# Distinct Patterns of Antibiotic Sensitivities in Ammonia‐Oxidising Archaea

**DOI:** 10.1111/1462-2920.70063

**Published:** 2025-03-11

**Authors:** Timothy Klein, Logan H. Hodgskiss, Max Dreer, J. Colin Murrell, Matthew I. Hutchings, Christa Schleper, Laura E. Lehtovirta‐Morley

**Affiliations:** ^1^ School of Biological Sciences University of East Anglia Norwich UK; ^2^ Department of Functional and Evolutionary Ecology, Archaea Biology and Ecogenomics Unit University of Vienna Vienna Austria; ^3^ School of Environmental Sciences University of East Anglia Norwich UK; ^4^ Department of Molecular Microbiology John Innes Centre Norwich UK

**Keywords:** ammonia‐oxidising archaea, antibiotics, genetic system, inhibition, selective enrichment

## Abstract

Ammonia‐oxidising archaea (AOA) are important microorganisms contributing towards the nitrogen flux in the environment. Unlike archaea from other major phyla, genetic tools are yet to be developed for the AOA, and identification of antibiotic resistance markers for selecting mutants is required for a genetic system. The aim of this study was to test the effects of selected antibiotics (hygromycin B, neomycin, apramycin, puromycin, novobiocin) on pure cultures of three well studied AOA strains, ‘*Candidatus* Nitrosocosmicus franklandianus C13’, *Nitrososphaera viennensis* EN76 and *Nitrosopumilus maritimus* SCM1. Puromycin, hygromycin B and neomycin inhibited some but not all tested archaeal strains. All strains were resistant to apramycin and inhibited by novobiocin to various degrees. As *N. viennensis* EN76 was relatively more resistant to the tested antibiotics, a wider range of concentrations and compounds (chloramphenicol, trimethoprim, statins) was tested against this strain. *N. viennensis* EN76 was inhibited by trimethoprim, but not by chloramphenicol, and growth recovered within days in the presence of simvastatin, suggesting either degradation of, or spontaneous resistance against, this compound. This study highlights the physiological differences between different genera of AOA and has identified new candidate antibiotics for selective enrichment and the development of selectable markers for genetic systems in AOA.

## Graphical abstract

Antibiotic sensitivity varies between different strains of ammonia‐oxidising archaea. The results of this study will be useful for selective enrichment and development of a genetic system for ammonia‐oxidising archaea.

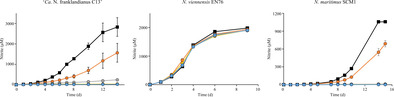

## 1 Introduction

Ammonia‐oxidising archaea (AOA) in the order Nitrososphaerales (formerly the phylum Thaumarchaeota and class Nitrososphaeria) play a crucial role in the cycling of nitrogen through their role in the oxidation of ammonia, the rate‐limiting step of nitrification (Brochier‐Armanet et al. 2008; Kerou et al. 2016; Rinke et al. 2021). AOA are chemoautotrophs, and in addition to nitrogen cycling, have important environmental functions in carbon cycling and production of vitamins (Könneke et al. 2014; Bayer et al. 2024). AOA are globally distributed in soils, and accounts for up to 40% of all prokaryotic cells in marine ecosystems (Karner et al. 2001; Gubry‐Rangin et al. 2011). Since their discovery, research efforts have aimed at characterising their physiology, biochemistry and genomic content. Furthermore, there is significant interest in understanding the contribution that AOA make to the nitrification process relative to their bacterial counterparts (e.g., ammonia‐oxidising bacteria [AOB]) (He et al. 2018; Yin et al. 2018; Rütting et al. 2021). Nevertheless, a major bottleneck in AOA research is the lack of a genetic system which would facilitate the characterisation of the molecular mechanisms dictating the physiology of these archaea.

A key component of a genetic system is the availability of a suitable selective agent which is essential for mutant selection. Antibiotic‐based selection has been instrumental in the development of archaeal genetic systems for lineages such as methanogens, halophiles and hyperthermophiles (Holmes et al. 1994; Liu et al. 2011; Nayak and Metcalf 2017; Deng et al. 2009). Therefore, insights into the antibiotic sensitivity patterns of the AOA, particularly pure cultures, will be beneficial towards the establishment of a genetic system.

Aside from their use as selective agents, antibiotics are also an important tool in archaeal research where they are used to aid the selective isolation of archaea from mixed microbial communities (Liu et al. 2019). Furthermore, as not usually pathogenic and therefore not historically the target of intense antibiotic research, the innate resistance of archaea to numerous antibiotics is exploited to differentiate between the biological activity of bacteria and archaea (Elevi Bardavid and Oren 2008; Taylor et al. 2010; Vajrala et al. 2014; Zhao et al. 2020). Archaea also represent unique models for testing new natural products because they have both eukaryotic and bacterial elements and may thus reveal new or unexpected mechanisms of action and targets (Williams et al. 2013).

Current knowledge of archaeal antibiotic sensitivity patterns has primarily been gleaned from studies on the major phyla Thermoproteota (formerly Crenarchaeota), Halobacteriota and Methanobacteriota (formerly Euryarchaeota). These studies have revealed that archaea are primarily sensitive to inhibitors of DNA and protein synthesis (Khelaifia and Drancourt 2012). Despite the discovery of AOA almost two decades ago, surprisingly little is known about their antibiotic sensitivity, with patterns mainly inferred from mixed cultures or enrichments (Schauss et al. 2009; Liu et al. 2019). These, however, can be misleading as the observed inhibition may result from indirect rather than direct inhibitory effects of the antibiotic (Woo et al. 2022). In addition, it is difficult to discern lineage‐specific sensitivity patterns among AOA from mixed communities, although they may have some practical applications in biased enrichments. To our knowledge, reports on the antibiotic sensitivity of pure cultures of AOA is restricted to a handful of strains and a limited number of compounds (Vajrala et al. 2014; Zhao et al. 2020). This limited knowledge on the antibiotic sensitivity of AOA is a bottleneck for establishing a genetic system for AOA, targeted enrichment strategies from environmental samples and identification of selective inhibitors.

In this study, antibiotics (hygromycin B, puromycin, neomycin, novobiocin) representing potential selective markers were tested against pure cultures of AOA. The antibiotics were selected based on five main criteria: (i) previously shown to exhibit anti‐archaeal activity, (ii) commercially available, (iii) antibiotics for which the genes conferring resistance are known, (iv) the stability of the antibiotics at the growth temperatures of the model AOA strains and (v) the antibiotic must be soluble in water (Table 1). In addition, apramycin, which has been used as a selective marker in bacterial genetics and which belongs to the same aminoglycosides class as hygromycin and neomycin, was included in the experiments to explore its potential use as an inhibitor of AOA. Due to *Nitrososphaera viennensis* EN76 being more resistant to the tested antibiotics compared to the other AOA strains, an extended range of concentrations and further antibiotics (chloramphenicol, trimethoprim, lovastatin, pravastatin, simvastatin) were screened against this strain to discover further suitable selective markers. The primary goal of this study was to identify antibiotics which inhibit pure cultures of the model AOA strains ‘*Candidatus* Nitrosocosmicus franklandianus C13’ (further referred to as *N. franklandianus*), *N. viennensis* EN76 and *Nitrosopumilus maritimus* SCM1, and to determine to which extent their inhibition thresholds vary between lineages.

**TABLE 1:** Selected antibiotics previously used for archaeal genetic systems and in archaeal research.

Antibiotic	Class	Structural subclass	Mode of action	Examples of established genetic systems	Used in AOA enrichments	References
Hygromycin B^a^	Aminoglycoside	Monosubstituted deoxystreptamine	Inhibition of protein synthesis	*Sulfolobus solfataricus*		Mann and Bromer (1958), Borovinskaya et al. (2008), Gritz and Davies (1983)
Apramycin^a^	Aminoglycoside					Pfister et al. (2003)
Neomycin^a^	Aminoglycoside	4,5 disubstituted deoxystreptamine	Inhibition of protein synthesis	*Methanococcus maripaludis*		Argyle et al. (1996)
Kanamycin	Aminoglycoside	4,6 disubstituted deoxystreptamine	Inhibition of protein synthesis		x	Recht and Puglisi (2001), Liu et al. (2019) and references therein
Gentamycin					x	Recht and Puglisi (2001), Liu et al. (2019) and references therein
Tobramycin						Liu et al. (2019)
Streptomycin	Aminoglycoside	Streptamine	Inhibition of protein synthesis		x	Recht and Puglisi (2001)
Puromycin^a^	Aminonucleoside		Inhibition of protein synthesis	*Methanosarcina acetivorans, Methanococcus voltae, Methanococcus maripaludis*		Nayak and Metcalf (2017), Bertani and Baresi (1987), Tumbula et al. (1994)
Novobiocin^a^	Aminocoumarin		Inhibition of DNA replication	*Haloferax strain* Aa 2.2	x	Holmes et al. (1994), Holmes and Dyall‐Smith (1990), Abby et al. (2018)
Trimethoprim^a^	Diaminopyrimidine		Inhibition of folate biosynthesis			Stuer‐Lauridsen and Nygaard (1998)
Chloramphenicol^a^	Phenicol		Inhibition of protein synthesis			Dridi et al. (2011)
Simvastatin^a^	Statin		Inhibition of archaeal membrane biosynthesis	*Sulfolobus islandicus* M.16.4, *Haloferax volcanii* DS2, *Thermococcus kodakaraensis* KOD1		Zhang and Whitaker (2012), Lam and Doolittle (1989), Matsumi et al. (2007)
Lovastatin^a^	Statin		Inhibition of archaeal membrane biosynthesis			Nkamga et al. (2017)
Pravastatin^a^	Statin		Inhibition of archaeal membrane biosynthesis	*Halorubrum lacusprofundi* ACAM34		Liao et al. (2016)

^a^ Antibiotics used in this study.

## 2 Experimental Procedures

### 2.1 Cultivation and Antibiotics Screening for AOA

Cultures were incubated static in the dark and pH was maintained between 7 and 8. All the AOA cultures tested in this study are pure cultures, including ‘*N. franklandianus*’. Batch cultures of ‘*N. franklandianus*’ and *N. viennensis* EN76 were routinely maintained in freshwater medium (FWM) (NaCl [1 g/L], MgCl_2_∙6H_2_O [0.4 g/L], CaCl_2_∙2H_2_O [0.1 g/L], KH_2_PO_4_ [0.2 g/L] and KCl [0.5 g/L]) supplemented with sterile stocks of modified trace element solution (1 mL/L), FeNaEDTA (1 mL/L), NaHCO_3_ (2 mM), vitamin solution (1 mL), HEPES buffer (10 mM) and phenol red at a final concentration of 1.4 μM. Vitamin solution consisted of biotin (0.02 g/L), folic acid (0.02 g/L), pyridoxine HCl (0.1 g/L), thiamine HCl (0.05 g/L), riboflavin (0.05 g/L), nicotinic acid (0.05 g/L), dl‐pantothenic acid (0.05 g/L), 4‐aminobenzoic acid (0.05 g/L), choline chloride (2 g/L) and vitamin B_12_ (0.01 g/L). As trimethoprim inhibits microorganisms by interfering with folic acid biosynthesis, the experiments with trimethoprim were carried out both in the presence and absence of the added vitamins. ‘*N. franklandianus*’ and *N. viennensis* EN76 cultures were supplied with NH_4_Cl at a final concentration of 5 and 3 mM, respectively, and *N. viennensis* EN76 with 0.5 mM sodium pyruvate. *N. maritimus* SCM1 was maintained in synthetic crenarchaeote medium (NaCl [26 g/L], MgCl_2_∙6H_2_O [5 g/L], MgSO_4_∙7H_2_O [5 g/L], CaCl_2_∙2H_2_O [1.5 g/L] and KBr [0.1 g/L]) supplemented with modified trace element solution (1 mL/L), FeNaEDTA (7.5 μM), NaHCO_3_ (2 mM), NH_4_Cl (1 mM), HEPES (10 mM) and KH_2_PO_4_ (2.9 mM). All glassware was acid‐washed in 10% (v/v) nitric acid prior to use and cultures were regularly monitored for the presence of heterotrophic bacteria by plating on LB plates and R2A agar plates. To test the effects of antibiotics, 500 mL ‘*N. franklandianus*’ cells were harvested during exponential phase (~0.7–1.2 mM nitrite (NO_2_^−^)) onto a 0.2‐μM pore size PES membrane filter (Millipore) under vacuum, washed and resuspended in FWM salts, and used as inoculum. *N. maritimus* SCM1 cultures were grown to mid‐late exponential phase (~200–500 μM NO_2_^−^), diluted 1:3 in 1× SCM salts and used as inoculum. *N. viennensis* cells were harvested from 400 to 500 mL of exponential phase culture by centrifugation at 5000 rpm for 30–40 min, resuspended and diluted in sterile FWM salts and used as inoculum. All assays were performed in 30‐mL plastic universal vials (Greiner Bio‐One) with 5 mL medium. Antibiotics were added to the desired concentration and the cultures were incubated at either 37°C (*N. viennensis* EN76 and ‘*N. franklandianus*’) or 28°C (*N. maritimus* SCM1). Antibiotics were added once in the beginning of the experiment. Minimum inhibitory concentration (MIC_95_, 95% inhibition) was defined as < 5% nitrite accumulation compared to the uninhibited control cultures at the end of each experiment.

### 2.2 Extended Measurements of *N. viennensis* EN76

For data shown in Figures 3 and 4, batch cultures of *N. viennensis* EN76 were grown in 30 mL polystyrene containers (Greiner Bio‐One) containing 20 mL growth medium with following differences to the above described medium: 2 mM NH_4_Cl, no phenol red, 1 mM pyruvate and 200 μg/mL kanamycin. Growth curves in Figures 3E and 4B–D were performed without the addition of vitamins (stocks grown without vitamins for multiple transfers before experiment). Tests were run in duplicates (trimethoprim, chloramphenicol, novobiocin, puromycin, lovastatin, pravastatin and simvastatin) at 42°C in the dark under static conditions. Antibiotics were added once cultures produced ~200 μM of nitrite. For antibiotics dissolved in DMSO (lovastatin and simvastatin) or ethanol (chloramphenicol) controls containing only solvent were included. No DMSO control was done for the pravastatin test (Figure 4D) and trimethoprim with vitamins (Figure 3E) since all cultures overlapped with normal controls. Additionally, an inhibitory concentration of simvastatin was added at various nitrite concentrations during growth to evaluate the effect of timing for the addition. A higher simvastatin stock solution negated the need for a DMSO control (Figure 4B).

## 3 Nitrite Measurements

Growth of AOA cultures was monitored by measuring nitrite accumulation. Nitrite concentration in the culture medium was measured using the Greiss colorimetric assay with sulphanilamide and *N*‐(1‐naphthyl) ethylenediamide as previously described in a 96‐well plate format as previously described (Lehtovirta‐Morley et al. 2016). Absorbance was measured at 540 nm using a VersaMax plate reader (Molecular Devices). The typical cell yields for the model organisms are 7.6 × 10^3^, 4 × 10^4^ and 5 × 10^4^ cells/nmol of nitrite accumulated, for ‘*N. franklandianus*’, *N. viennensis* EN76 and *N. maritimus* SCM1, respectively (Lehtovirta‐Morley et al. 2016; Tourna et al. 2011; Könneke et al. 2005).

## 4 Results and Discussion

All experiments were conducted with the three strains ‘*N. franklandianus*’, isolated from neutral pH agricultural soil (Lehtovirta‐Morley et al. 2016), *Nitrososphaera viennensis* EN76 (*N. viennensis*) isolated from neutral pH garden soil (Stieglmeier et al. 2014) and *Nitrosopumilus maritimus* SCM1 (*N. maritimus*) from a marine aquarium (Könneke et al. 2005). Growth of strains was followed through the production of nitrite in cultures, which has been confirmed to parallel cell numbers in growing cultures for all strains (Lehtovirta‐Morley et al. 2016; Stieglmeier et al. 2014; Könneke et al. 2005).

### 4.1 Sensitivity of AOA to Aminoglycosides

**FIGURE 1:**
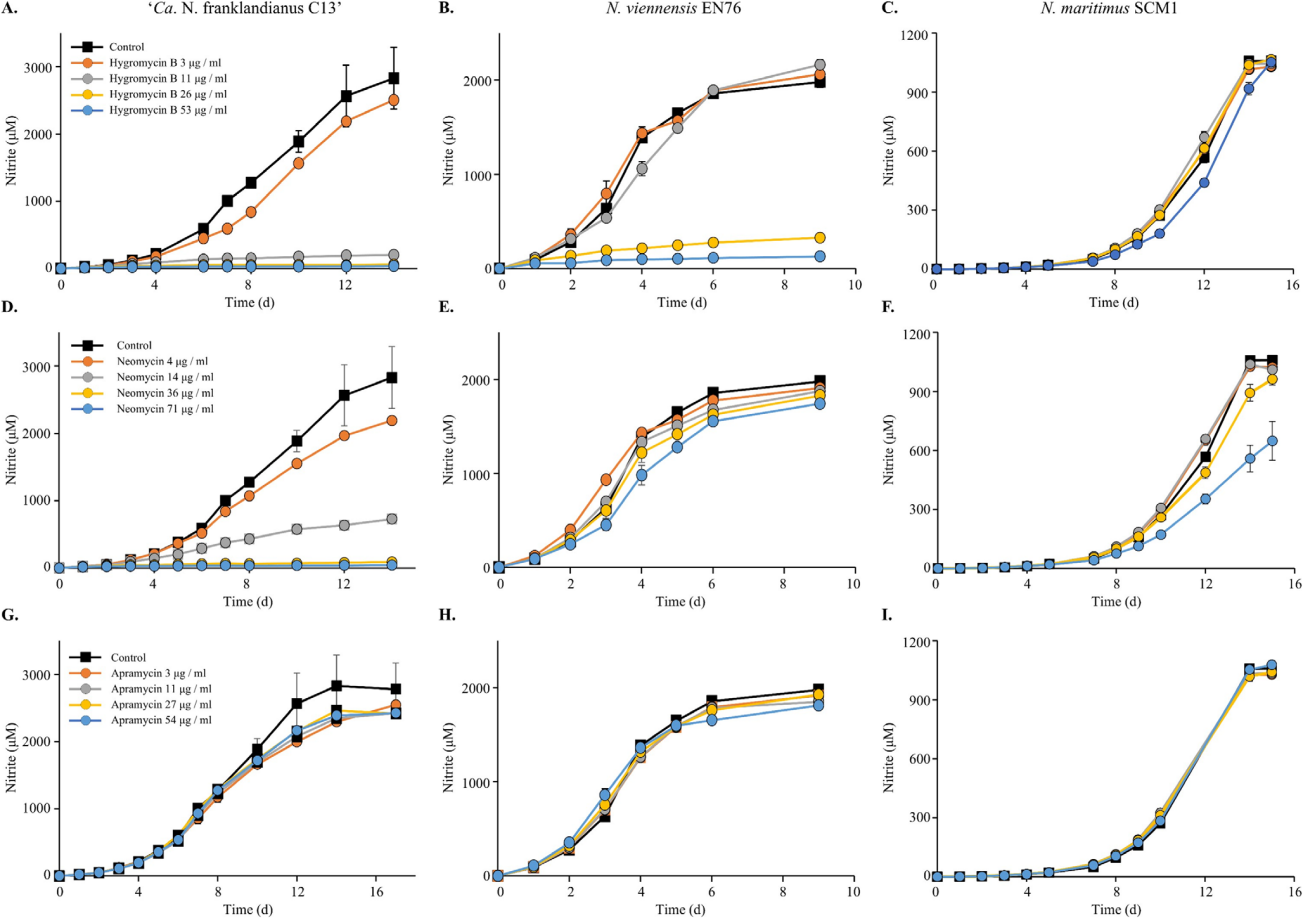
In vivo antibiotic sensitivity of ‘*Ca*. *N. franklandianus* C13’, *N. viennensis* EN76, and *N. maritimus* SCM1, to hygromycin B (A–C), neomycin (D–F) and apramycin (G–I). Inhibition of nitrite (NO_2_^−^) production was assumed to indicate sensitivity. All antibiotic‐treated cultures were compared to untreated cultures as a control. The nitrite concentrations plotted represent the average of three replicate cultures and control cultures were not treated with any antibiotics. Error bars represent the standard error of mean (SEM).

Aminoglycoside antibiotics target the bacterial 30S ribosomal subunit and inhibit the translation of bacterial mRNAs into proteins, typically leading to truncated peptides. The aminoglycosides hygromycin B, neomycin and apramycin were screened against the model AOA *N. franklandianus*, *N. viennensis* and *N. maritimus*. Hygromycin B strongly inhibited nitrite production, which is used as a proxy for growth, by *N*. *franklandianus* at a concentration of 11 μg/mL, and completely inhibited by > 26 μg/mL (Figure 1A) (Table 2). On the other hand, *N. viennensis* and *N. maritimus* were less sensitive to hygromycin B, and although *N. viennensis* was > 80% inhibited by concentrations of > 26 μg/mL, neither of these model AOA were fully inhibited by the concentrations of hygromycin B tested in this experiment (Figure 1B,C). Neomycin inhibited *N. franklandianus* completely at > 36 μg/mL (Figure 1D) (Table 2) but, as observed with hygromycin B, neither *N. viennensis* nor *N. maritimus* was fully inhibited by the presence of neomycin (Figure 1E,F). The tested concentrations of neomycin had no effect on the nitrite accumulation by *N. viennensis* and only partially inhibited *N. maritimus* (Figure 1E,F). In contrast to hygromycin B and neomycin, apramycin had no observable effect on the growth of any of the AOA strains tested in this study (Figure 1G–I).

**TABLE 2:** Minimum inhibitory concentrations (MIC_95_) (μg/mL) of the antibiotics in ammonia oxidising archaea.

Organism	Hygromycin B	Neomycin	Apramycin	Antibiotics	Novobiocin	Chloramphenicol	Trimethoprim	Simvastatin	Statins	Pravastatin
				Puromycin					Lovastatin	
‘*Ca*. Nitrosocosmicus franklandus C13’	26	36	> 54	27	> 63	—	—	—	—	—
*Nitrososphaera viennensis* EN76	> 53	> 600 (> 71)^a^	> 54	(> 54)	> 400 (> 63)^a^ ^,^ ^b^	> 150^a^	> 10^a^	> 10^a^ ^,^ ^c^	> 20^c^	> 8.5^c^
*Nitrosopumilus maritimus* SCM1	> 53	> 71	> 54	11	> 63	—	—	—	—	—

^a^ Duplicates.

^b^ MIC_95_ missed by 0.12% (2 μM NO_2_^−^).

^c^ Spontaneous resistance occurred.

Aminoglycosides can be categorised into four structural subclasses based on the presence of an aminocyclitol ring (commonly 2‐deoxystreptamine, 2‐DOS): (i) streptidine‐containing (streptomycin), (ii) monosubstituted deoxystreptamine (e.g., hygromycin B, apramycin), (iii) 4,5 disubstituted deoxystreptamine or 4,5 2‐DOS (e.g., neomycin) and (iv) 4,6 disubstituted deoxystreptamine or 4,6 2‐DOS (e.g., kanamycin, gentamycin) (Krause et al. 2016) (Table 1). *N. maritimus* was resistant to both monosubstituted aminoglycosides tested while *N. franklandianus* and *N. viennensis* were only sensitive to hygromycin B. All three strains responded to neomycin, albeit to varying degrees, the only representative of the 4,5 2‐DOS tested in this study. Interestingly, aminoglycosides from the 4,6 disubstituted 2‐DOS (kanamycin) and streptidine class (streptomycin) are generally inactive against AOA and are often used in AOA enrichments (Lehtovirta‐Morley et al. 2014, 2016; Stieglmeier et al. 2014; Abby et al. 2018; Bayer et al. 2019). Resistance of AOA to 4,6 2‐DOS aminoglycosides might be expected based on the widespread use of this structural class in AOA enrichments and axenic cultures. However, it was recently shown that tobramycin, which belongs to the same structural class, significantly reduced the abundance of AOA in an enrichment (Liu et al. 2019). The resistance to apramycin of all three strains highlights this antibiotic as a suitable choice for AOA enrichments but not as a potential selection agent.

### 4.2 Sensitivity to Puromycin

**FIGURE 2:**
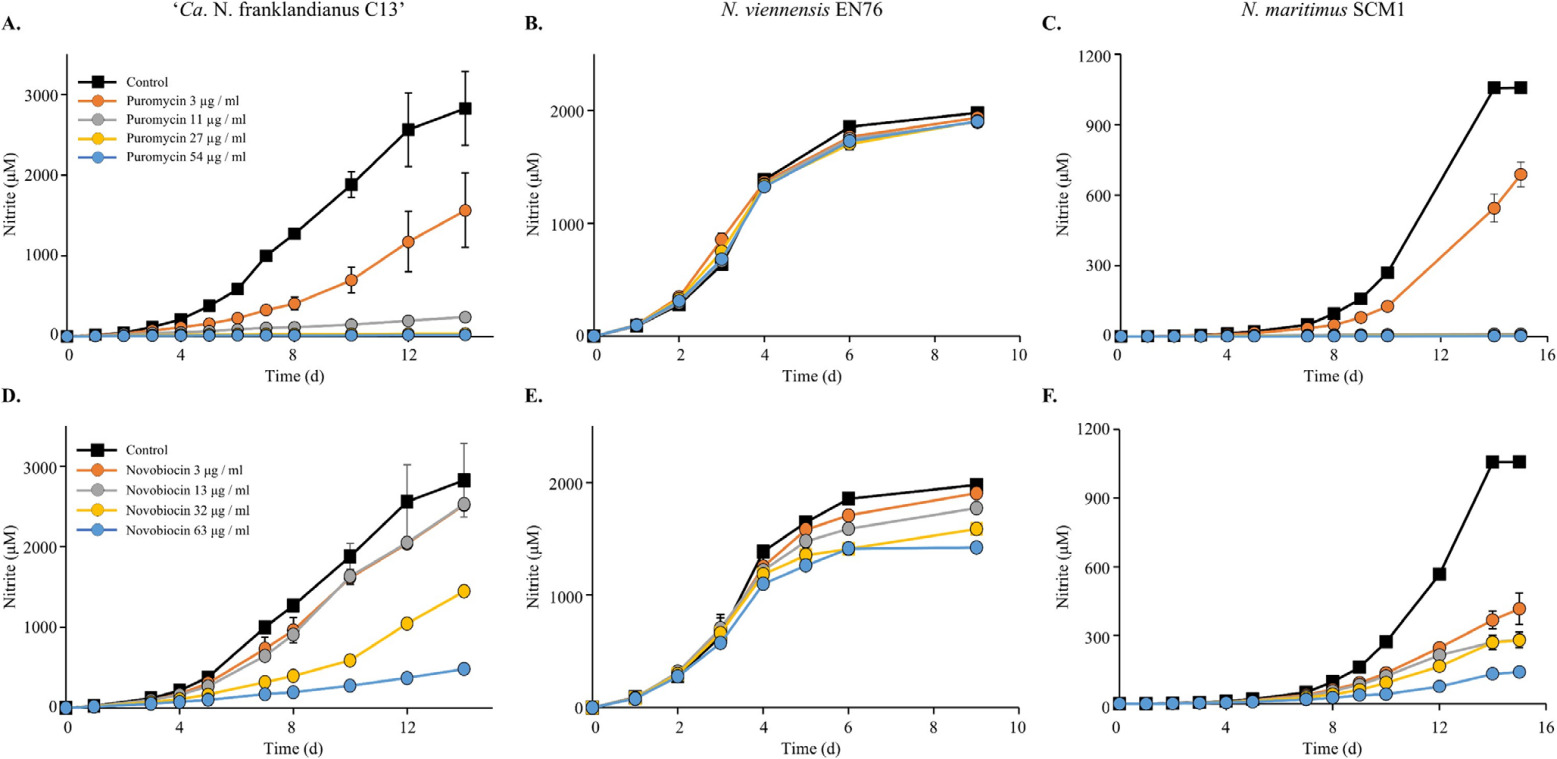
In vivo antibiotic sensitivity of ‘*Ca*. *N. franklandianus* C13’, *N. viennensis* EN76, and *N. maritimus* SCM1, to puromycin (A–C) and novobiocin (D–F). Inhibition of nitrite (NO_2_^−^) production was assumed to indicate sensitivity. All antibiotic‐treated cultures were compared to untreated cultures as a control. The nitrite concentrations plotted represent the average of three replicate cultures and control cultures were not treated with any antibiotics. Error bars represent the standard error of mean (SEM).

Puromycin is an aminonucleoside, a class of antibiotics which inhibit translation in some archaea, bacteria and eukaryotes. Puromycin was chosen because it is used as a selection agent in genetic systems of methanogenic archaea (Gernhardt et al. 1990; Patel et al. 1994; Argyle et al. 1996; Nayak and Metcalf 2017) (Table 1). Puromycin was strongly inhibitory to Nitrosocosmicus franklandianus (Figure 2A). Even the lowest puromycin concentration tested, 3 μg/mL, resulted in 45% inhibition of nitrification in Nitrosocosmicus franklandianus, and growth was completely inhibited by 27 μg/mL puromycin (Table 2). In contrast to Nitrosocosmicus franklandianus, *N. viennensis* was resistant to puromycin at all the tested concentrations and the highest tested concentration of puromycin, 54 μg/mL had no effect on this strain (Figure 2B). The marine strain *N. maritimus* was strongly inhibited by puromycin, with concentrations ≥ 11 μg/mL inducing complete inhibition relative to the control cultures (Figure 2C).

### 4.3 Sensitivity to Topoisomerase Inhibitors

The aminocoumarin antibiotic, novobiocin, is a topoisomerase inhibitor that specifically targets DNA gyrase (Serizawa et al. 2010). It has recently been established that DNA gyrase is absent from the order Nitrososphaerales (Villain et al. 2022) but novobiocin inhibited *N. franklandianus* cultures (Figure 2D). Although the inhibition of *N. franklandianus* was not complete, a concentration of 63 μg/mL resulted in > 80% decrease in nitrite production (Figure 2D). *N. maritimus* was also sensitive to all tested concentrations of novobiocin although even the highest concentration (63 μg/mL) did not fully inhibit growth (Figure 2F). Although *N. viennensis* was inhibited by novobiocin to a lesser extent than the other tested AOA, there was a 28% reduction in the final nitrite yield compared to the uninhibited control in the presence of 63 μg/mL novobiocin (Figure 2E). In *N. maritimus* and *N. franklandianus*, both growth rate and yield were reduced, which is a typical pattern of inhibition by antibiotics. The sensitivity to novobiocin by all three AOA strains is surprising and may indicate the presence of a novel target(s) for novobiocin in these archaea.

Novobiocin targets the ATPase domain of DNA gyrase subunit B (GyrB) which is situated within a structural region known as the Bergerat fold (Maxwell and Lawson 2003). This Bergerat fold is also found in other ATPase‐containing proteins such as Hsp90, MutL and even histidine kinases (Dutta and Inouye 2000). The only type I topoisomerase that is ATP‐dependent is reverse gyrase, but this is exclusively present in hyperthermophilic archaea and bacteria (Schoeffler and Berger 2008). Thus, a likely target for novobiocin in the AOA strains would be a type II topoisomerase due to the presence of an ATP‐binding site. A plausible candidate for novobiocin inhibition in all three AOA strains would therefore be topoisomerase VI. This topoisomerase is widely distributed in the archaea, including those that contain DNA gyrase (Garnier et al. 2021), and it is the only representative of the type IIB topoisomerases (Champoux 2001; Corbett and Berger 2003). Topoisomerase VI contains a Bergerat fold but previous in vitro tests on topoisomerase VI from *Sulfolobus shibatae*, showed that this enzyme is insensitive to novobiocin (Bergerats et al. 1994). The authors reported that novobiocin was stable for at least 20 min at 80°C, and thus, the high temperatures at which these assays were performed cannot explain the lack of inhibition. It is also possible that novobiocin is targeting other Bergerat fold‐containing proteins such histidine kinases which are abundantly present in the genomes of the three AOA strains. Interestingly, the sensitivity to novobiocin does not seem to be a general trait among all AOA. For example, an enrichment of the thermophilic AOA strain ‘*Ca*. Nitrosocaldus cavascurensis’ exhibited no response to treatment with 100 μg/mL of novobiocin (Abby et al. 2018).

Taking the data for all three tested AOA into account, the observed antibiotic sensitivity patterns were distinct between the strains. Furthermore, the data indicate that AOA are sensitive to certain aminoglycosides (hygromycin B and neomycin), an aminocoumarin (novobiocin) and an aminonucleoside (puromycin) (Table 2).

### 4.4 Identification of Further Selective Markers for *N. viennensis*

**FIGURE 3:**
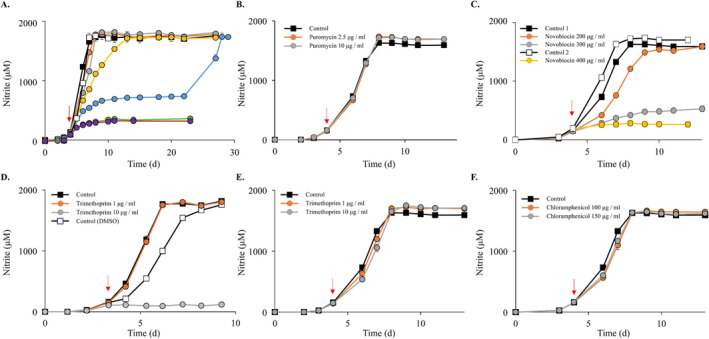
The effects of an extended range of concentrations and types of antibiotics on *N. viennensis* EN76. (A) Neomycin, (B) puromycin, (C) novobiocin, (D) trimethoprim in medium without vitamins (DMSO), (E) trimethoprim in medium with vitamins (DMSO), (F) chloramphenicol (H_2_O). The red arrow denotes when the antibiotics were added. All experiments were performed in duplicate. Error bars represent standard error and may be smaller than the marker. Key for (A) neomycin: black squares = Control 1, orange circles = 50 μg/mL, grey circles = 100 μg/mL, yellow circles = 150 μg/mL, blue circles = 300 μg/mL, white squares = Control 2, green circles = 400 μg/mL, purple circles = 600 μg/mL. Concentrations 50–300 μg/mL neomycin were performed in parallel with Control 1, and concentrations 400–600 μg/mL neomycin with Control 2. For (C) concentrations 200–300 μg/mL novobiocin were performed in parallel with Control 1, and concentration 400 μg/mL novobiocin with Control 2.

The screening of the three model AOA (Figures 1 and 2) identified that *N. viennensis* was resistant to all but one (hygromycin B, Figure 1B) of the tested antibiotics at the concentrations used in the experiments. To expand the range of suitable selective markers for this strain, some of the promising antibiotics (those inhibitory to both *N. franklandianus* and *N. maritimus*) were tested at higher concentrations and additional compounds (trimethoprim, chloramphenicol and statins) were included in the growth experiments. Increasing the concentration of neomycin and novobiocin resulted in near‐complete inhibition of nitrite accumulation by *N. viennensis*, with the most inhibited cultures accumulating 228 and 75 μM nitrite, respectively, after the addition of antibiotics (Figure 3A,C). Neomycin treatments at 400 and 600 μg/mL were monitored for 23 days, and it is not known if the nitrification would have eventually recovered at these concentrations during prolonged incubation as it did with 300 μg/mL neomycin (Figure 3A). For novobiocin, the effect was very close to the 95% inhibition and novobiocin could be a candidate compound for future studies where selective inhibition is required (Figure 3C). In addition, the puromycin screening was repeated to test whether incubation temperature or the time of antibiotic addition could affect the results. Puromycin was added at concentrations of up to 10 μg/mL using this experimental setup in *N. viennensis* (Figure 3B), which confirmed that neither changing the incubation temperature from 37°C to 42°C nor the time of introducing antibiotics affected the results. Whether higher puromycin concentrations can inhibit *N. viennensis* would be an interesting topic for future studies.

Trimethoprim interferes with folate biosynthesis by competitively inhibiting dihydrofolate reductase and preventing conversion of dihydrofolate to tetrahydrofolate, and chloramphenicol is a protein synthesis inhibitor. Although neither chloramphenicol nor trimethoprim has been used for selection in archaeal genetic systems previously, the resistance genes are known. Furthermore, we hypothesised that they could be suitable inhibitors for AOA because *N. viennensis*' genome encodes for a predicted dihydrofolate reductase family protein (NVIE_011950), and, for example, the halophilic archaeon *Haloferax volcanii* is inhibited by 30 μM (~8.7 μg/mL) trimethoprim and several methanogenic archaeal strains by < 25 μg/mL chloramphenicol (Dridi et al. 2011; Stuer‐Lauridsen and Nygaard 1998). As hypothesised, 10 μg/mL trimethoprim was inhibitory to *N. viennensis* in the vitamin‐free medium, and the inhibitory effect of trimethoprim was not observed in the medium supplemented with vitamins (Figure 3E). This strongly suggests that trimethoprim inhibits *N. viennensis* by specifically interfering with vitamin biosynthesis.

Contrary to our expectations, chloramphenicol (< 150 μg/mL) had no effect on the growth of *N. viennensis* (Figure 3F). Investigating the full inhibitory range of chloramphenicol was complicated by the low solubility of chloramphenicol in water and the necessity to introduce another solvent. Ethanol was used as a solvent for higher concentrations, but ethanol itself was inhibitory to the growth, making it difficult to assess the effects of chloramphenicol at concentrations of 200 μg/mL and above (Figure S1).

### 4.5 Sensitivity to Statins

**FIGURE 4:**
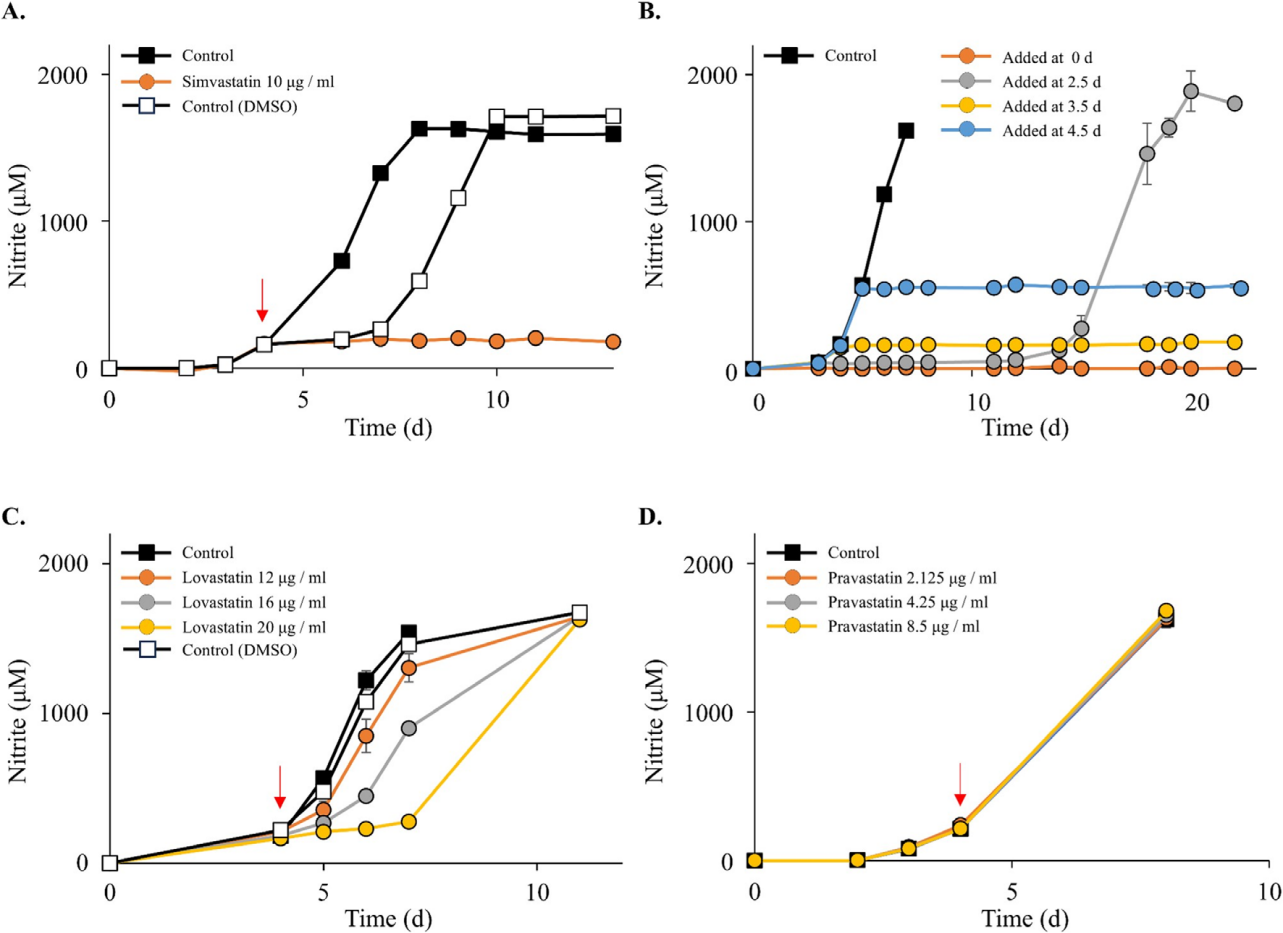
The effects of statins on *N. viennensis* EN76. (A), simvastatin (B) simvastatin (resistance), 10 μg/mL simvastatin was added at different timepoints, (C) lovastatin, (D) pravastatin. Red arrow denotes when the statins were added. All experiments were performed in duplicate. Error bars represent standard error and may be smaller than the marker.

To explore other suitable inhibitory compounds for *N. viennensis*, the effect of statins (lovastatin, pravastatin, simvastatin) on growth was investigated. Statins interfere with lipid biosynthesis by inhibiting hydroxymethylglutaryl‐CoA reductase and have been widely used in archaeal research for genetic systems in both Thermoproteota and Halobacteriota, and for selective inhibition (Zhao et al. 2020; Zhang and Whitaker 2012; Lam and Doolittle 1989). A concentration of 10 μg/mL (23.9 μM) was chosen as this has previously been used successfully in *Sulfolobus islandicus* (now *Saccharolobus islandicus*) (Zhang and Whitaker 2012). Nitrite accumulation by *N. viennensis* initially ceased after introduction of 10 μg/mL (23.9 μM) simvastatin (Figure 4A). This is in conflict with previous results that show a concentration above 32 μM was needed for inhibition (Zhao et al. 2020). However, subsequent tests in medium lacking vitamins showed growth in some cultures at around 15 days irrespective of the time of simvastatin addition (Figure 4B). While the growth conditions were slightly different in this study, it calls into question the reliability of simvastatin as a selection marker in AOA. The unexpected recovery of nitrite accumulation may be either due to spontaneous resistance arising in *N. viennensis* or breakdown of simvastatin. Two further statins, lovastatin and pravastatin, were tested against *N. viennensis*, but neither compound was fully inhibitory at the tested range, < 20 and < 8.5 μg/mL, respectively (Figure 4C,D).

Stability of simvastatin is greater in acidic pH and low temperatures (Alvarez‐Lueje et al. 2005). Nevertheless, simvastatin has been successfully used for selection in the thermophiles *S. islandicus* and *Thermococcus kodakaraensis*, which are routinely grown at temperatures > 78°C (Zhang and Whitaker 2012; Matsumi et al. 2007). Given that *N. viennensis* was grown at 42°C and pH 7.5, only slightly higher than the pH typically used for cultivating *Thermococcus*, simvastatin may be expected to be relatively stable in the growth conditions used in this study. A selection system based on statin inhibition relies on the overexpression of the *hmg* *A* gene to overcome statin sensitivity. It is therefore not surprising that resistance has been seen in other archaeal studies (Zhang and Whitaker 2012; Zheng et al. 2012; Matsumi et al. 2007; Thiel et al. 2014; Farkas et al. 2012). Additionally, while most archaeal species used with simvastatin are heterotrophic or mixotrophic, AOA like *N. viennensis* are strictly autotrophic and are assumed to have a tight regulatory control over metabolites feeding the central carbon metabolism from the carbon fixation cycle. While most carbon is assumed to leave the 3‐hydroxybutyrate/4‐hydroxypropionate cycle via succinyl‐CoA, the cycle is replenished by acetyl‐CoA (Estelmann et al. 2011; Könneke et al. 2014). An interference with the HmgA protein would likely lead to an accumulation of acetyl‐CoA and a regulatory response in *N. viennensis*. This, along with the extended generation time of *N. viennensis* compared to other archaea and the inconsistent response of *N. viennensis* to simvastatin concentrations, may preclude the effectiveness of statins as a selection marker for AOA. Curiously, the recovery from simvastatin treatment was observed only in the treatment where simvastatin was added after 2.5 days, which may suggest that breakdown of simvastatin is not responsible for the observed recovery (Figure 4B). A more thorough analysis of different simvastatin concentrations and the occurrence of spontaneous resistance would be needed to accurately compare to other archaeal species and to resolve the variability observed in *N. viennensis*.

### 4.6 Applications and Impacts of Antibiotics for Studying AOA

The potent inhibition of some AOA by puromycin and hygromycin B highlights these antibiotics as potential selection agents against *N. franklandianus* (Figure 1). Puromycin is typically used at concentrations ranging between 2 and 10 μg/mL in methanogen genetics. In bacterial models such as *Mycoplasma* sp., the *pac* gene (encoding for the puromycin *N*‐acetyltransferase) is capable of conferring resistance to the bacterium *M. capricolum* up to 500 μg/mL of puromycin (Algire et al. 2009). This would be sufficient for future genetic transformation experiments, considering that *N. franklandianus* was strongly inhibited by > 27 μg/mL of puromycin. Hygromycin B has been useful in genetic studies in *Sulfolobales* (Atomi et al. 2012) and an advantage of hygromycin B as a selection agent is the availability of a thermotolerant resistance gene encoding hygromycin phosphotransferase (*hpt*) (Cannio et al. 2001) Typical concentrations of hygromycin B used for *S. solfataricus*, an important genetic model for the hyperthermophilic archaea, are approximately 300 μg/mL (Cannio et al. 2001) In bacterial models such as *Escherichia coli*, hygromycin B is used at concentrations exceeding 140 μg/mL (Kalivoda et al. 2011). These concentrations far exceed the concentrations needed to strongly inhibit *N. franklandianus* (~11 μg/mL) indicating that this strain is highly sensitive to this antibiotic and thus hygromycin B seems like a suitable candidate in a selection system.

On the other hand, it was challenging to find a good selective marker for *N. viennensis*. This strain was more resistant to the tested antibiotics than *N. franklandianus* and *N. maritimus*. Novobiocin is one of the more promising selective compounds for inhibiting *N. viennensis*. However, since DNA gyrase is absent, novobiocin presumably exerts its inhibitory effect through an alternative, currently unknown target, which may complicate the choice of resistance genes required for a genetic system. Trimethoprim may be a more suitable selective marker, as this study demonstrated that trimethoprim is inhibitory to *N. viennensis* in the vitamin‐free medium only. This suggests that trimethoprim acts through the same mechanism as it does in other microorganisms and interferes with vitamin biosynthesis. The effect of statins, especially the inconsistent observed resistance to simvastatin, was unexpected in *N. viennensis*. If organisms can degrade simvastatin or spontaneously develop resistance to it, or if simvastatin is not stable for a sufficiently long duration in the cultivation conditions of AOA, it may limit the uses of simvastatin as a selective agent against AOA.

Different sensitivity patterns of AOA may have some practical applications. For example, during isolation efforts and maintenance of pure cultures, it is commonplace to use a cocktail of antibiotics to retard bacterial growth. This work identified an additional antibiotic, apramycin, that does not inhibit AOA but has been shown to be highly effective against gram‐negative bacteria (Bordeleau et al. 2021). Since all known bacterial nitrifiers are gram‐negative, apramycin presents a possible selective inhibitor of AOB. However, since selective inhibitors are typically desired when studying mixed communities (e.g., microcosms), the efficacy of apramycin in this context would need to be tested.

In addition to using antibiotics for selective enrichment of cultures and for the development of genetic tools, it is plausible that antibiotics would influence archaeal communities in the environment. Neomycin, apramycin and novobiocin are used in veterinary medicine for treatment of bacterial infections, and introduction of aminoglycosides from antibiotic‐treated animals into soil ecosystems is a recognised concern (Coates et al. 2022). In addition, neomycin has been detected in wastewater treatment plants (Stenholm et al. 2022), and many antibiotics, including novobiocin and aminoglycosides, are produced by soil bacteria. Statins are widely used as cholesterol‐lowering medicines and have been detected in both wastewater treatment plants and in freshwater environments (Santos et al. 2016). If archaea reside in environments in the vicinity of animals and are exposed to these compounds, there is a possible impact on the abundance, diversity and function of the archaea, as there is on bacteria. In addition, AOA are found in wastewater treatment plants and in freshwater habitats, and it is plausible that they would come into contact with statins and aminoglycosides in these environments.

### 4.7 Potential Mechanisms and Limitations in Interpreting the Response of AOA to Antibiotics

The results in this study clearly indicate that the antibiotic sensitivity patterns differ between phylogenetically and physiologically distinct AOA strains. Although determining a robust lineage‐specific sensitivity pattern will require testing more strains and compounds, the observation that AOA exhibit differing sensitivity patterns will need to be strongly considered when selecting the antibiotics for enrichment of these archaea. There are several factors which may affect the sensitivity of AOA to antibiotics, including but not limited to, cell envelope architecture and permeability, growth conditions such as biofilm, presence or absence of a target within the cell and intrinsic antibiotic resistance mechanisms.

An example of a physiological distinction that may contribute towards contrasting sensitivities is the outer cell envelope. It is an important cellular structure that controls the influx and efflux of different molecules and acts as a molecular sieve (von Kügelgen et al. 2024). While *N. franklandianus* is surrounded by an uncharacterised cell wall (Nicol et al. 2019), *N. viennensis* and *N. maritimus* are both enveloped by an S‐layer (Stieglmeier et al. 2014; Qin et al. 2017). *N. maritimus* has P6 S‐layer symmetry which is common among methanogenic and halophilic archaea (Rodrigues‐Oliveira et al. 2017). It is therefore interesting that this strain shares antibiotic sensitivity patterns with several methanogenic and halophilic strains which are sensitive to puromycin but are only mildly or completely unaffected by hygromycin B (Possot et al. 1988; Mondorf et al. 2012; Nayak and Metcalf 2017). In comparison, *N. viennensis* shares antibiotic sensitivities and P3 S‐layer symmetry with other archaea such as the *Sulfolobales* (Cannio et al. 1998, 2001). Whether S‐layer symmetry influencing antibiotic sensitivity, and the role of transporters, require investigation using various methods in the future.

An additional key difference to consider between the three strains is the growth medium. While the two soil AOA strains grow in virtually identical growth media, *N. maritimus* requires a growth medium with a higher salt concentration and ionic strength. These conditions have previously been reported to influence the activity of aminoglycoside antibiotics (Coronado et al. 1995). The authors demonstrated in 13 halophilic bacterial strains that when the salt concentration in the medium was lowered from 10% (w/v) to 1% (w/v), the minimal inhibitory concentration of the aminoglycoside antibiotics decreased. The authors similarly demonstrated this effect in *E. coli* . It is possible that the salt concentration may play a role in sensitivity to aminoglycosides in other AOA strains, although exposing microorganisms to osmotic conditions below optimum could also plausibly affect the cell wall and induce stress.

## 5 Summary and Future Prospects

The primary aim of this study was to find suitable selective agents against AOA for the establishment of a genetic system. Puromycin and hygromycin B were both identified as potentially promising candidates for which resistance markers are available. This makes these two antibiotics attractive candidates for a selection system. It will, however, be necessary to determine whether puromycin and hygromycin B resistant mutants evolve under selective pressure and how frequently such spontaneous mutants arise. Furthermore, it is evident that AOA differ in their sensitivities to antibiotics, and this may have some useful practical applications such as intentionally introducing bias into enrichment cultures. With the exception of novobiocin, trimethoprim and simvastatin, all of the antibiotics that inhibited one or more AOA strains are classified as protein synthesis inhibitors belonging to either the aminoglycosides or aminonucleosides. Interestingly, one of the few previously studied AOA inhibitors, cycloheximide, is also a protein synthesis inhibitor.

The mechanisms and nature (biological vs. abiotic) of the observed simvastatin resistance in *N. viennensis* are unclear and will need further investigation. A low rate of mutation is very important for *N. franklandianus* cells as it takes approximately 2 months for visible colonies of *N. franklandianus* to develop on solid medium (Klein et al. 2022). During the experiments with puromycin, no spontaneous resistance was observed in *N. franklandianus*, which is crucial for the robustness of any future genetic system using puromycin‐based selection. Furthermore, to determine the molecular targets for novobiocin, biochemical and structural studies will need to be conducted. Future studies should explore the mechanisms of the observed patterns of inhibition, and could use transcriptomic and proteomic approaches in addition to biochemical and structural methods. Nevertheless, puromycin, hygromycin B and trimethoprim were successfully identified as selective agents for at least one of the model AOA in this study, and the resistance genes for all of these compounds are known. This means that although many questions remain open with regard to the mechanisms of action, the identification of suitable antibiotics will enable us to proceed with the development of genetic systems in AOA.

## Author Contributions

**Timothy Klein:** conceptualization, methodology, investigation, writing – original draft, formal analysis, writing – review and editing. **Logan H. Hodgskiss:** conceptualization, methodology, investigation, formal analysis, writing – review and editing. **Max Dreer:** conceptualization, methodology, investigation, formal analysis, writing – review and editing. **J. Colin Murrell:** conceptualization, funding acquisition, writing – review and editing, supervision. **Matthew I. Hutchings:** conceptualization, funding acquisition, writing – review and editing, supervision. **Christa Schleper:** conceptualization, funding acquisition, writing – review and editing, supervision. **Laura E. Lehtovirta‐Morley:** conceptualization, funding acquisition, supervision, writing – original draft, writing – review and editing.

## Conflicts of Interest

The authors declare no conflicts of interest.

## Supporting information


**Figure S1.** The inhibition of *N. viennensis* EN76 by ethanol. Due to the low solubility of chloramphenicol, ethanol was tested as an alternative solvent. Red arrow denotes when ethanol or chloramphenicol dissolved in ethanol was added. The experiment was performed in duplicate. Error bars represent standard error and may be smaller than the marker.

## Data Availability

Data are available from the corresponding author upon reasonable request.
