# Supplementary figures and images for: Distinct Patterns of Antibiotic Sensitivities in Ammonia‐Oxidising Archaea
Source: Environ Microbiol. 2025 Mar 11;27(3):e70063. doi: 10.1111/1462-2920.70063 (PMC11897584; doi:10.1111/1462-2920.70063)

**Appendix**


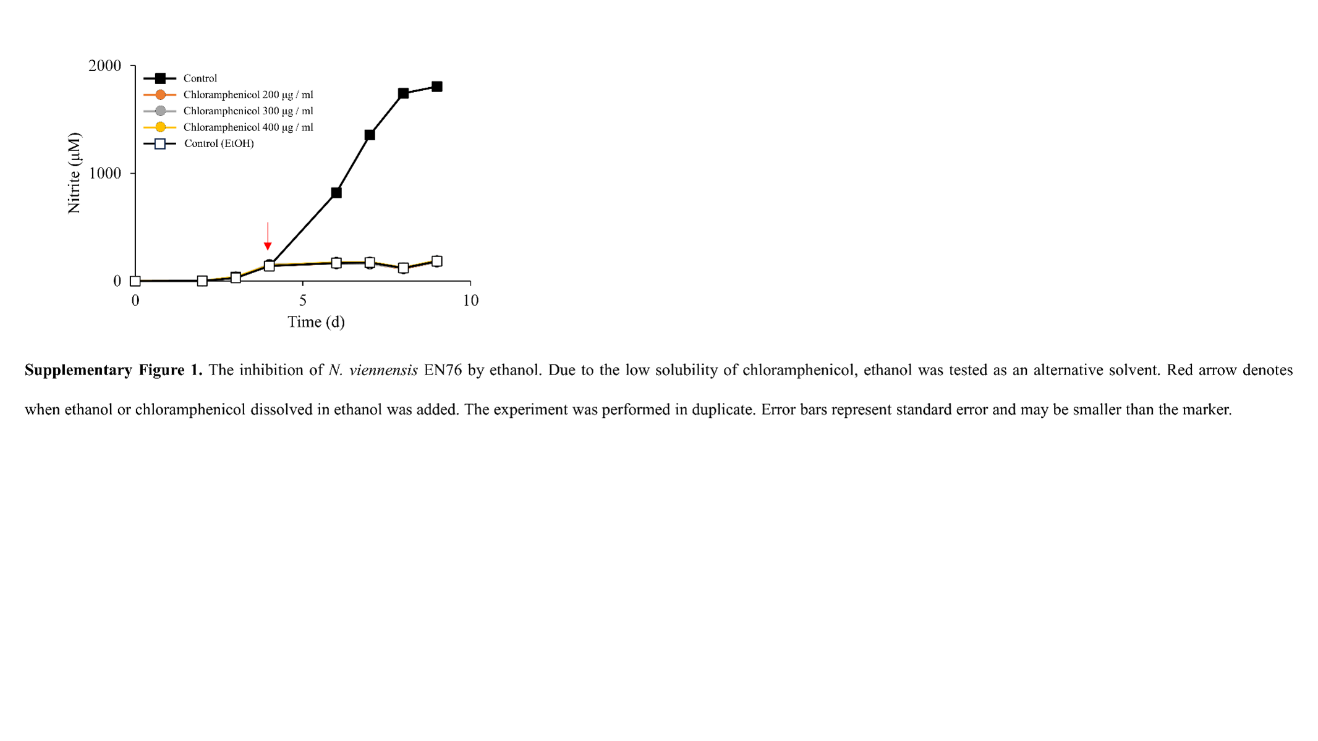

Supplement: Supplementary file 1 — Figure S1. The inhibition of N. viennensis EN76 by ethanol. Due to the low solubility of chloramphenicol, ethanol was tested as an alternative solvent. Red arrow denotes when ethanol or chloramphenicol dissolved in ethanol was added. The experiment was performed in duplicate. Error bars represent standard error and may be smaller than the marker. [file EMI-27-e70063-s001.docx]
